# Supplementary material for: Long-Term Analysis of Pertussis Vaccine Immunity to Identify Potential Markers of Vaccine-Induced Memory Associated With Whole Cell But Not Acellular Pertussis Immunization in Mice
Source: Front Immunol. 2022 Feb 8;13:838504. doi: 10.3389/fimmu.2022.838504 (PMC8861382; doi:10.3389/fimmu.2022.838504)
Supplement: Supplementary file 1 [file DataSheet_1.pdf]

Supplementary Material

1 Supplementary Data

Table S1. Immunized mice have significantly elevated serum titer levels compared to mock-vaccinated animals. Statistics shown for **(A)** anti-*B. pertussis* serum titers **(B)** anti-FHA serum titers, **(C)** and anti-pertussis toxin serum titers. Days 20 and 22 were from three independent experiments (n=12). Day 35 is from four independent experiments (n=16). Days 60 and 90 were from two independent experiments (n=8). Days 152 (n=4), 365 (n=8), and 532 (n=4) are from one independent experiment. The *p*-values were calculated for each time point using ANOVA followed by a Tukey's multiple-comparison test, *p < 0.05, **p < 0.01, ***p < 0.001, ****p < 0.0001. Error bars are mean ± SEM values (n=4-16), aP (n=4-16), and wP (n=4-16).

	**Comparison**	**20**	**22**	**35**	**60**	**90**	**152**	**365**	**532**
**A**	PBS vs aP	****	****	****	****	****	****	****	****
	PBS vs wP	****	****	****	****	****	****	****	****
	aP vs wP	NS	NS	NS	NS	NS	NS	NS	NS
**B**	**Comparison**	**20**	**22**	**35**	**60**	**90**	**152**	**365**	**532**
	PBS vs aP	****	****	****	****	****	****	****	****
	PBS vs wP	****	****	****	****	****	****	****	****
	aP vs wP	**NS**	**NS**	**NS**	*	*	**NS**	**NS**	**NS**
**C**	**Comparison**	**20**	**22**	**35**	**60**	**90**	**152**	**365**	**532**
	PBS vs aP	****	****	****	****	****	****	****	****
	PBS vs wP	**NS**	**NS**	**NS**	**NS**	**NS**	**NS**	**NS**	**NS**
	aP vs wP	****	****	****	****	****	****	****	****

Table S2. Flow cytometry panel design (**A**) T_{FH} cells and (**B**) *B. pertussis*$^{+}$ MBCs.

A

**T Follicular Helper Cells**			
**Antibody**	**Fluorophore**	**Company**	**Catalog Number**
CD185	PE	BD Biosciences	551959
PD-1	PerCP-eFluor710	eBioscience	46-9985-82
CD4	APC-Cy7	BioLegend	100526
CD3ε	BV510	BD Biosciences	563024

B

***B.pertussis*$^{+}$ Memory B Cells**			
**Antibody/dye**	**Fluorophore**	**Company**	**Catalog Number**
IgG	Alexa Fluor 488	Invitrogen	A-11059
CD38	Pe-Vio770	Miltenyi Biotec	130-109-336
CD80	BV421	BD Biosciences	562611
CD3ε	BV510	BD Biosciences	563024
CD45R	APC-Cy7	BD Biosciences	552094
Dye	*Bac*Light Red	Invitrogen	B35001

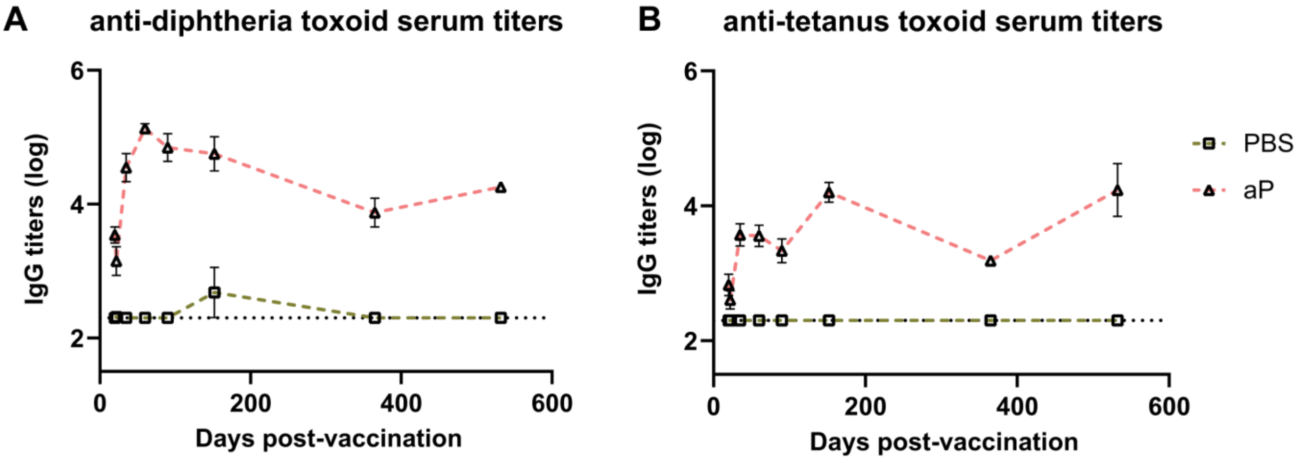

Comparison	20	22	35	60	90	152	365	532
PBS vs aP	****	****	****	****	****	***	****	****

Comparison	20	22	35	60	90	152	365	532
PBS vs aP	***	*	****	****	****	****	****	***

Figure S1. Anti-diphtheria and anti-tetanus antibodies persist as far as day 532 post-prime without waning. **(D)** IgG anti-diphtheria toxoid antibodies in non-challenged, vaccinated mice (n=4-16) and **(E)** IgG anti-tetanus toxoid antibodies measured in blood serum collected at day 20, day 22, day 35, day 60, day 90, day 152, day 365, and day 532 post-vaccination. Antibody responses were at or below the limit of detection in PBS vaccinated mice. Data were log10-transformed. Days 20 and 22 were from three independent experiments (n=12). Day 35 is from four independent experiments (n=16). Days 60 and 90 were from two independent experiments (n=8). Days 152 (n=4), 365 (n=8), and 532 (n=4) are from one independent experiment. The *p*-values were calculated for each time point using ANOVA followed by a Tukey's multiple-comparison test, $*p < 0.05$, $***p < 0.001$, $****p < 0.0001$. Error bars are mean ± SEM values.

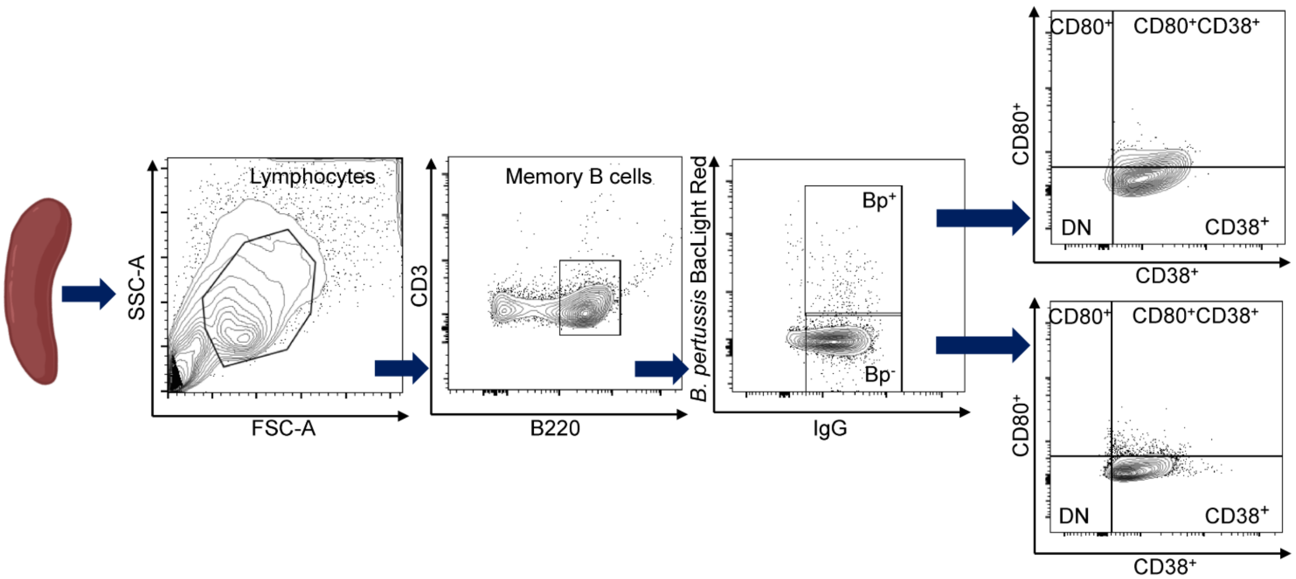

Figure S2. Flow cytometry allows for detection of *B. pertussis*$^{+}$ MBCs. After selection of MBCs using our protocol and Miltenyi memory B cell isolation kit reagents, single live cells were gated to select for lymphocytes. From the lymphocyte population gating selected for CD3^{-} B220^{+} (CD45R) cells. From the B220^{+} (CD45R) population we gated to select *B. pertussis*$^{+}$ cells ultimately isolating the *B. pertussis*$^{+}$ MBC population. We further analyzed this population looking at CD38^{+}, CD80^{+}, CD38^{+}CD80^{+} and double negative populations
